# The Epidemic among Cattle

**Published:** 1860-06

**Authors:** 


*The Epidemic among Cattle.*—The legislature of Massachusetts has appointed a commission of three persons to investigate the character of the apparently contagious disease which is now spreading with great fatality among the herds throughout the country. The State is to assume the cost of the investigation, paying to the owners the value of cattle which are to be slaughtered, under the direction of the committee, on the appearance of the disease. The pathological appearances of animals which were killed as soon as affected, or which died from the disease, are very indefinite; but it appears to be a disease of the lungs, and the first symptoms are said to be a croup-like respiration.—*Medical & Surgical Reporter.*

